# Complete resection of a giant mediastinal paraganglioma in the aortopulmonary window using aortic root transection: Report in a patient with the Carney triad

**DOI:** 10.1016/j.xjtc.2026.102398

**Published:** 2026-04-27

**Authors:** Lucas Kostic, Charbel Al Zreibi, Amedeo Iafaldano, Charles-Henri Gautier, Laure Gibault, Kim-Diep Dang-Tran, Marco Alifano, Paul Achouh, Francoise Le Pimpec-Barthes

**Affiliations:** a Department of Thoracic Surgery and Lung Transplantation, Hôpital Européen Georges-Pompidou, Assistance Publique-Hôpitaux de Paris, Université Paris-Cité, Paris, France; b Department of Cardiovascular Surgery, Hôpital Européen Georges-Pompidou, Assistance Publique-Hôpitaux de Paris, Université Paris-Cité, Paris, France; c Department of Pathology, Hôpital Européen Georges-Pompidou, Assistance Publique-Hôpitaux de Paris, Université Paris Cité, Paris, France; d Department of Radiology, Hôpital Européen Georges-Pompidou, Assistance Publique-Hôpitaux de Paris, Université Paris-Cité, Paris, France; e Department of Thoracic Surgery, Hôpital Cochin Assistance Publique-Hôpitaux de Paris, Université Paris Cité, Paris, France

**Figure:**
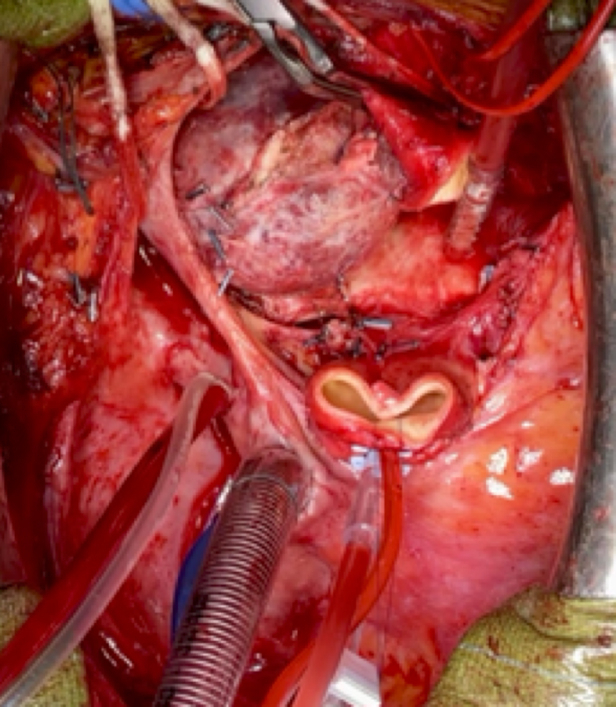
Transected aorta exposing the paraganglioma in the aortopulmonary window.

**Central Message**

Temporary aortic transection enables safe and complete resection of a large aortopulmonary window paraganglioma.

The Carney triad is a rare tumor syndrome associated with extra-adrenal paragangliomas, pulmonary chondromas, and gastric stromal tumors.^1^ Mediastinal paragangliomas are uncommon and may become symptomatic when compressing adjacent vascular or airway structures. Their diffuse hypervascularity from various mediastinal structures and their proximity to major organs make surgical resection particularly challenging. We report a large symptomatic paraganglioma of the aortopulmonary window in a patient with the Carney triad that required resection under cardiopulmonary bypass using temporary transection of the ascending aorta—a technique not previously described, to our knowledge, for this condition.

## Case Presentation

**Figure 1:**
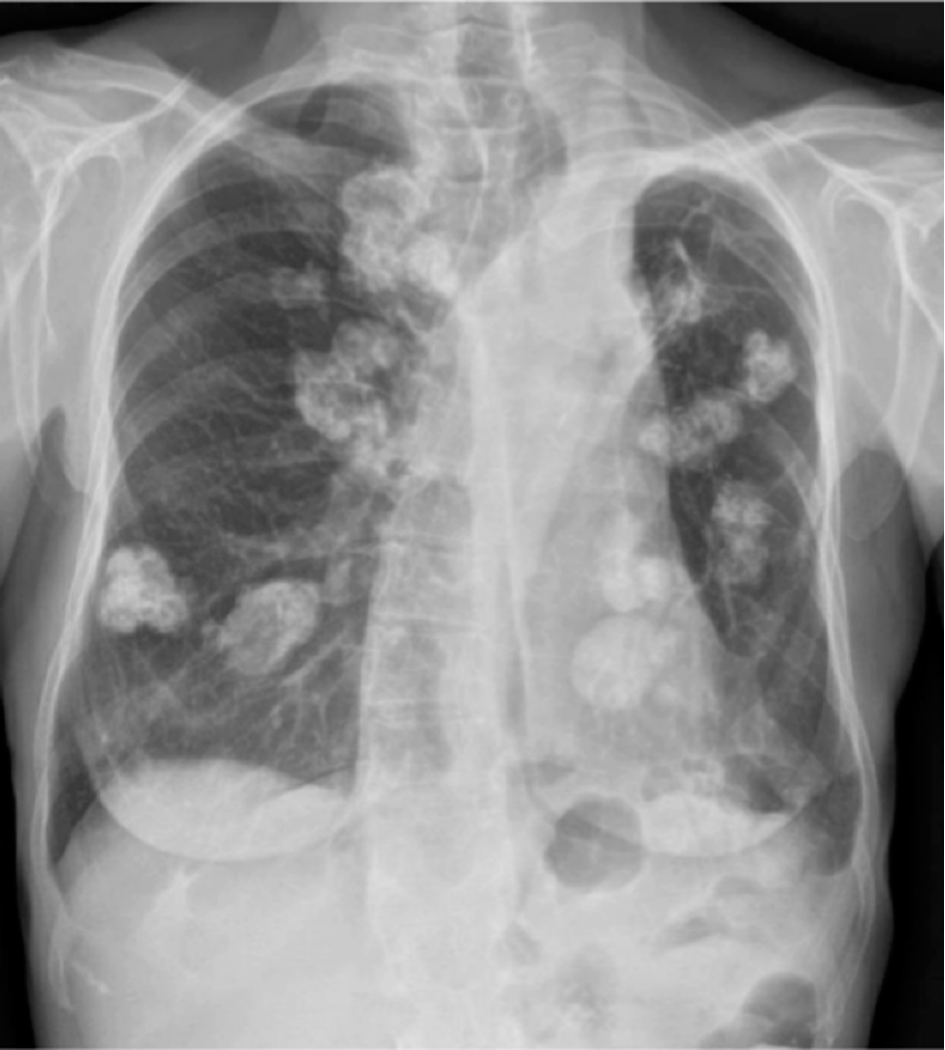
Preoperative radiograph of the chest showing the presence of multiple bilateral pulmonary chondromas.

A 53-year-old woman with the Carney triad and a pathogenic succinate dehydrogenase mutation was referred for a progressively enlarging mediastinal paraganglioma. At 17 years of age, she underwent subtotal gastrectomy for a gastric stromal tumor. Over the following years, she developed multiple bilateral pulmonary chondromas (Figure 1) that initially were mistaken for metastases but later confirmed by thoracic biopsy. When the patient was 24 years of age, recurrent hemoptysis required 2 pulmonary resections through left thoracotomy.

When the patient was 31 years of age, positron emission tomography revealed 2 carotid body lesions and a 29-mm paraganglioma in the aortopulmonary window. Embolization was attempted but did not alter the tumor's progression, and the patient was subsequently monitored. Over time, she developed 13 calcified pulmonary nodules, several increasing in size, and new esophageal and gastric lesions treated endoscopically. Cervical paragangliomas remained stable.

**Figure 2:**
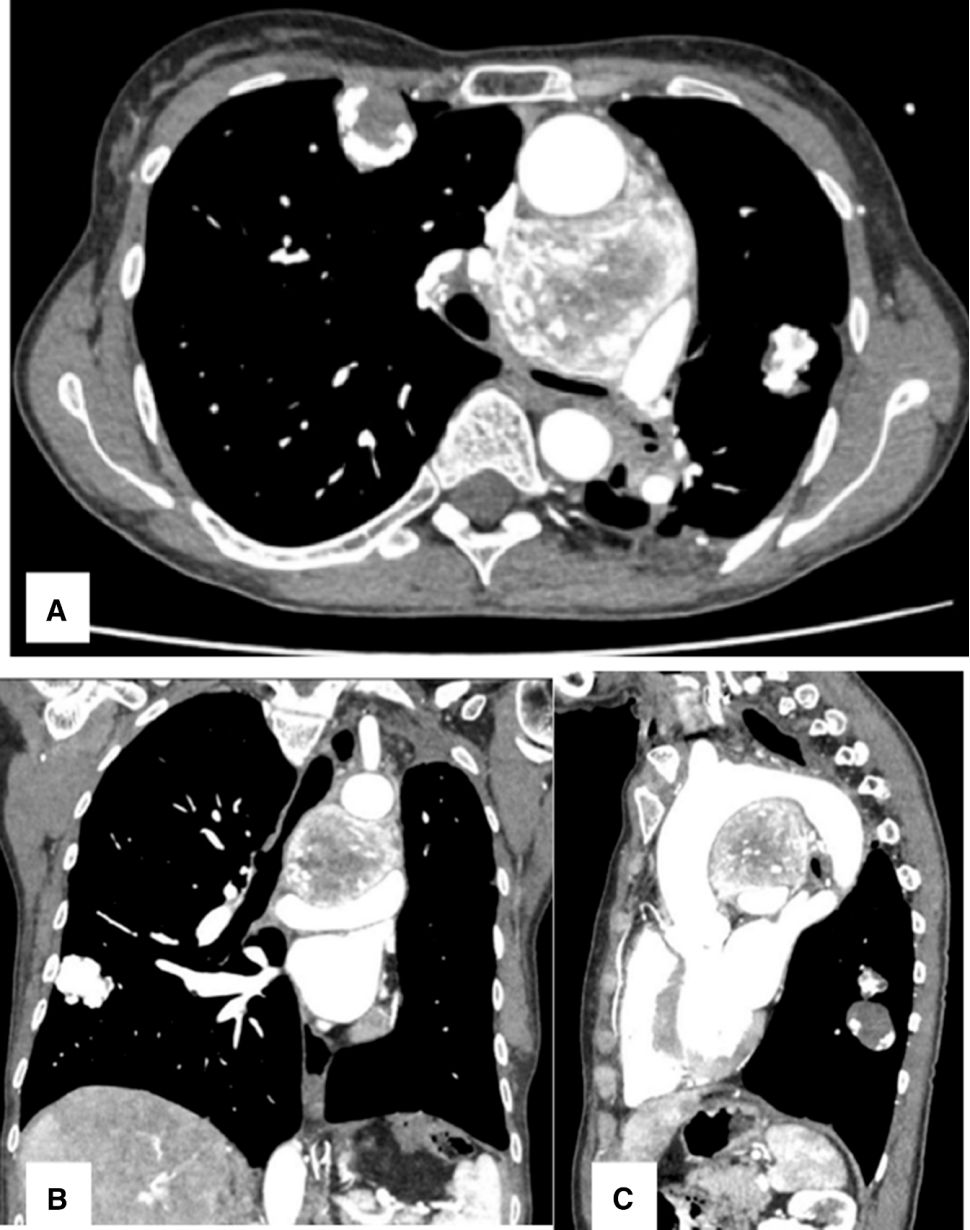
A-C, Contrast-enhanced preoperative computed tomography scan of the chest in sagittal, coronal, and axial views showing a mass of the aortopulmonary window measuring 65 × 60 × 52 mm, located posterior to the ascending aorta, below the aortic arch, above the right pulmonary artery, medial to the superior vena cava, and anterior to the carina and the left main bronchus.

The mediastinal paraganglioma enlarged to 65 mm, causing dysphonia, exertional dyspnea, and recurrent pneumonia attributable to airway compression (Figure 2, *A* and *B*). Computed tomography demonstrated close contact with the ascending aorta, carina, and pulmonary artery roof, without clear evidence of invasion (Figure 2, *C*). The lesion was nonsecreting. Findings on coronary angiography were normal, and pulmonary function tests showed preserved respiratory capacity. Given symptomatic progression and tumor enlargement, surgical resection under cardiopulmonary bypass with temporary ascending aortic transection was decided after a technical meeting comprising expert surgeons.

## Operative Technique

A midline sternotomy was performed. After the pericardium was opened, the tumor was mobilized from the ascending aorta. Given significant vascularity and bleeding risk, cardiopulmonary bypass was initiated via right femoral arterial cannulation and right atrial drainage.

**Figure 3:**
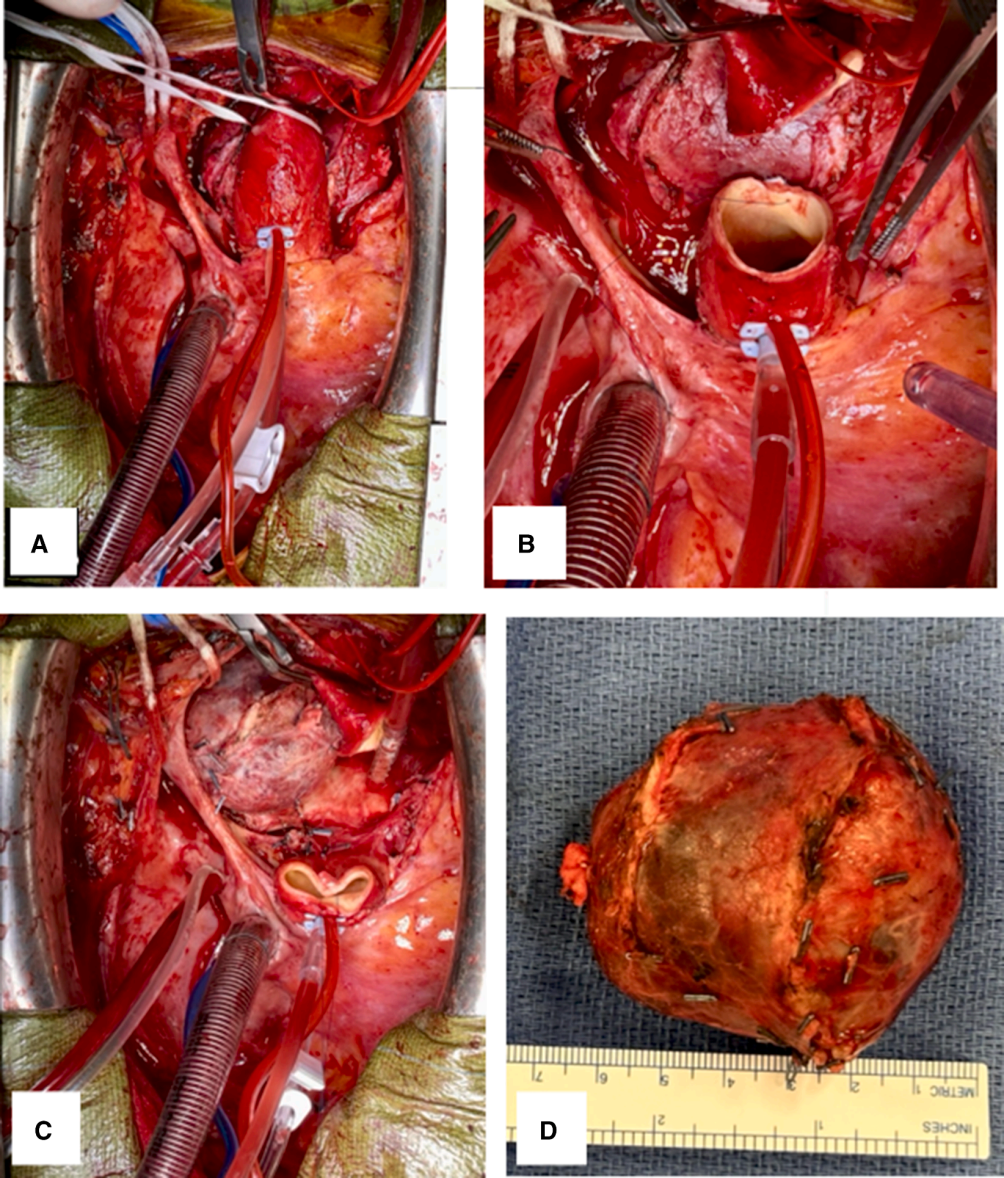
Intraoperative views after median sternotomy. A, Aortic crossclamping after placement of cardiopulmonary bypass cannulas (one in the right femoral artery for arterial inflow and one through the right atrial appendage for venous drainage). B, Transection of the ascending thoracic aorta exposing the paraganglioma located in the aortopulmonary window. C, Tumor dissection with release of the roof of the pulmonary artery and the left main bronchus. D, Paraganglioma after extraction.

The mass was dissected from the superior vena cava and pulmonary artery trunk. A vent tube was placed in the right superior pulmonary vein. The aorta was crossclamped, and antegrade cold crystalloid cardioplegia was administered (Figure 3, *A*). The ascending aorta was then transected transversely just above the coronary ostia (Figure 3, *B*), providing wide exposure of the posterior mediastinum, particularly the roof of the right pulmonary artery and the left mainstem bronchus (Figure 3, *C*). This approach allowed safe control of the tumor's posterior and inferior attachments. A complete en bloc resection was achieved (Figure 3, *D*).

**Figure 4:**
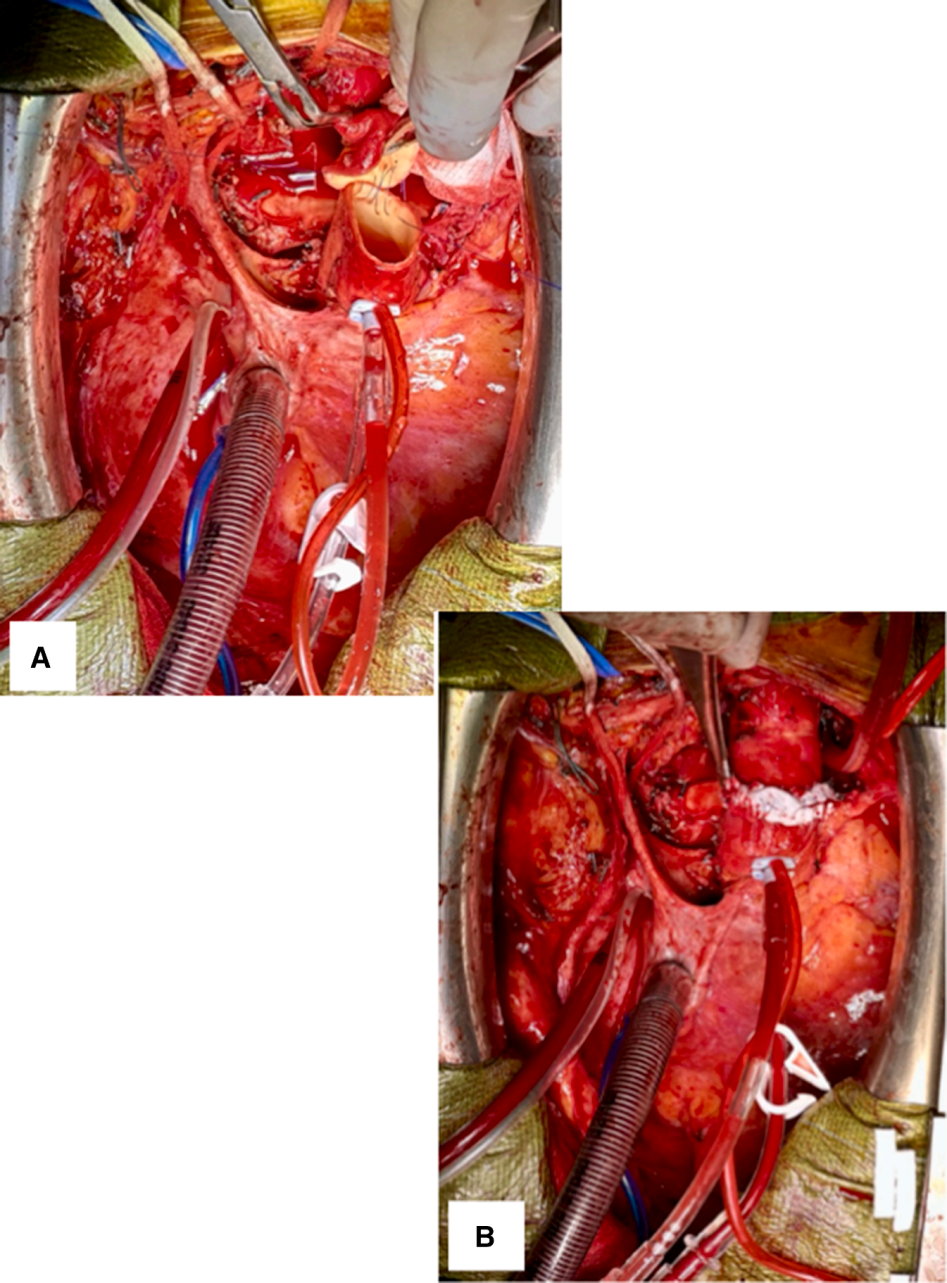
End-to-end anastomosis of the 2 aortic edges. A, Posterior wall suturing using a running suture reinforced with pledgetted U sutures. B, Anterior wall suturing using a running suture reinforced with a Teflon felt.

A termino-terminal aortic anastomosis was performed using a running suture. As usually done in our practice, the posterior wall was reinforced using U pledgetted sutures, and the anterior wall was reinforced with a Teflon felt (Figure 4, *A* and *B*). After deairing and unclamping, the heart resumed sinus rhythm with stable hemodynamics, and weaning from bypass was uncomplicated. Total ischemia and bypass times were 32 and 110 minutes, respectively. Hemostasis was achieved, and the sternum closed in standard fashion.

## Results

**Figure 5:**
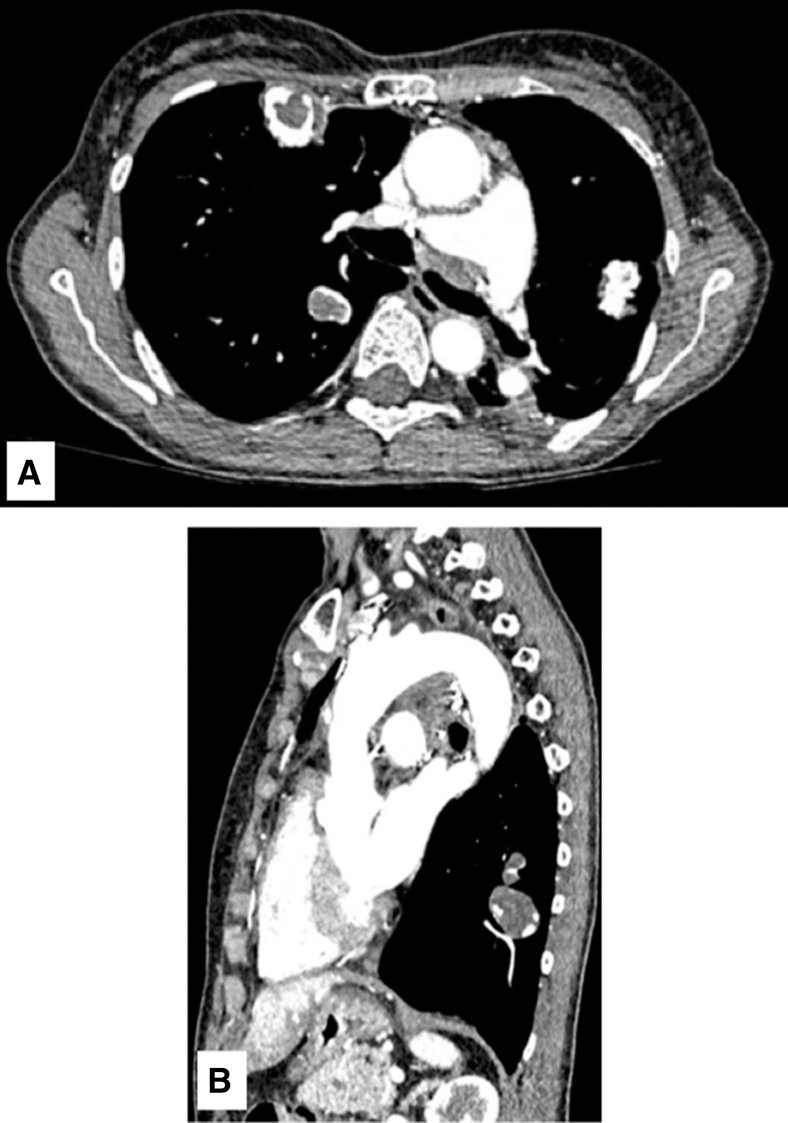
Contrast-enhanced computed tomography scan of the chest at 10 months postoperatively in axial (A) and sagittal (B) views showing the tumorectomy site with restoration of the normal anatomy of the aortopulmonary window.

Postoperative recovery was uneventful. Echocardiography showed preserved ventricular function and normal aortic flow. Histopathology confirmed paraganglioma, with a Pheochromocytoma of the Adrenal gland Scaled Score of 9 and a Grading of Adrenal Pheochromocytoma and Paraganglioma Score of 5, indicating moderate differentiation and potential aggressiveness. Resection margins were clear. Postoperative computed tomography demonstrated restoration of normal aortopulmonary anatomy and resolution of airway compression (Figure 5, *A* and *B*). At 12-month follow-up, the patient remained free of recurrence. A mild left vocal cord hypomobility improved with speech therapy. The patient provided written informed consent for the publication of her case and anonymized data, given its extreme rarity.

## Discussion

This case demonstrates that temporary ascending aortic transection under cardiopulmonary bypass can provide excellent exposure for safe resection of large paragangliomas in the aortopulmonary window. To our knowledge, this is the first reported case of this technique in the context of the Carney triad.

Mediastinal paragangliomas represent fewer than 0.3% of mediastinal tumors and are extremely rare in the aortopulmonary window. They may be secreting or nonsecreting and although typically benign, carry risks of recurrence, local invasion, and resistance to chemoradiotherapy. Preoperative embolization may reduce bleeding,^2^ but results are inconsistent. In our patient, previous embolization did not influence growth, illustrating the uncertain role of interventional radiology in syndromic disease. Complete surgical resection without fragmentation, at risk of severe bleeding, remains the recommended treatment whenever feasible.^3^

Surgical difficulty is related to hypervascularity coming from the various mediastinal structures and proximity to major vessels and airways. Cardiopulmonary bypass offers hemodynamic stability, reduces tumor perfusion, and facilitates dissection in high-risk regions.^4^ In this case, aortic transection provided unparalleled exposure of the posterior mediastinum, enabling safe control of the pulmonary artery and airway structures.^5^ Moreover, if there had been tumor invasion requiring segmental aortic resection, this could have been done safely using this surgical strategy.

An important lesson concerns timing. Progressive compression led to significant respiratory symptoms. Earlier intervention for enlarging paragangliomas in the aortopulmonary window may prevent morbidity and simplify surgery. Because evidence remains limited, further studies are needed to define optimal timing in the Carney triad.

## Conclusions

Temporary aortic transection under cardiopulmonary bypass can allow safe, complete resection of large mediastinal paragangliomas in the Carney triad. Early surgical intervention should be considered for enlarging aortopulmonary window lesions to prevent symptomatic airway and vascular compression.

### Declaration of Generative AI and AI-Assisted Technologies in the Writing Process

During the preparation of this work the authors used ChatGPT (OpenAI) in order to perform language editing. After using this tool/service, the authors reviewed and edited the content as needed and take full responsibility for the content of the publication.

## Conflict of Interest Statement

The authors reported no conflicts of interest.

The *Journal* policy requires editors and reviewers to disclose conflicts of interest and to decline handling or reviewing manuscripts for which they may have a conflict of interest. The editors and reviewers of this article have no conflicts of interest.
